# Editorial: Genomic alteration landscapes of aging, metabolic disorders, and cancer: Emerging overlaps and clinical importance

**DOI:** 10.3389/fgene.2022.1102953

**Published:** 2023-01-06

**Authors:** Jaspreet Kaur Dhanjal, Rajkumar Singh Kalra, Dhanendra Tomar, Amrendra K. Ajay

**Affiliations:** ^1^ Department of Computational Biology, Indraprastha Institute of Information Technology Delhi, New Delhi, India; ^2^ Immune Signal Unit, Okinawa Institute of Science and Technology Graduate University, Okinawa, Japan; ^3^ Department of Internal Medicine, Section of Cardiovascular Medicine, Section of Molecular Medicine, Wake Forest University School of Medicine, Winston-Salem, NC, United States; ^4^ Department of Medicine, Harvard Medical School, Boston, MA, United States; ^5^ Division of Renal Medicine, Department of Medicine, Brigham and Women’s Hospital, Boston, MA, United States

**Keywords:** genetic alterations, cancer, aging, metabolic disorder, mutations, senescence

**FIGURE 1:**
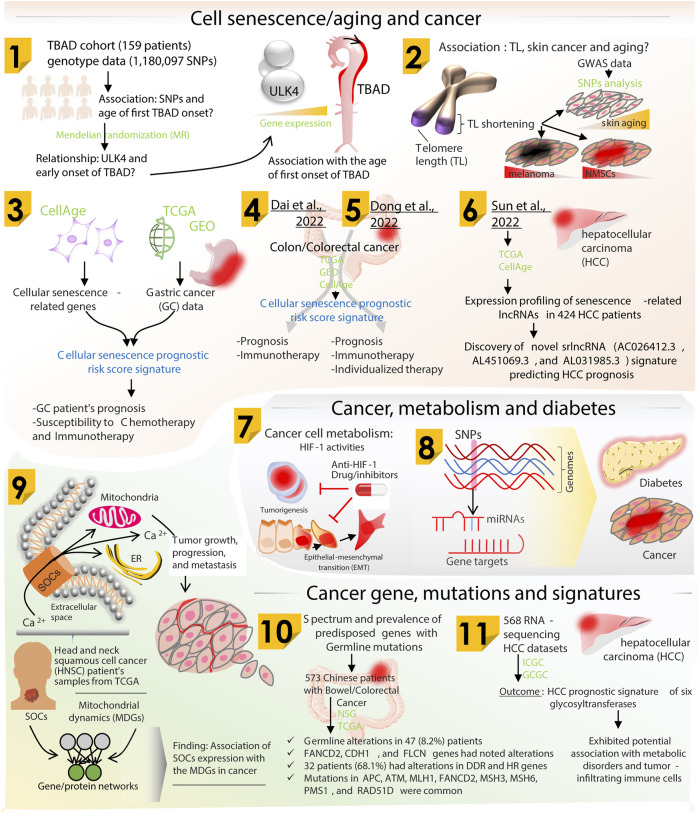
Schematic diagram showing research summarized majorly on three themes including cell senescence/aging and cancer, metabolism and cancer gene mutations/signatures. Research Topic articles are marked by serial number within the related schematic section.

The biology of aging, cancer, and various metabolic disorders shows a clear association with genetic and epigenetic changes. These genomic alterations arise from diverse intrinsic and extrinsic/environmental factors. The efficiency of a cell to proofread its newly synthesized DNA strand gradually decreases with age hampering its genomic integrity. An increased burden of genomic changes, therefore, gives rise to multiple health issues like metabolic disorders (Abou Ziki and Mani, 2016; Varshavi et al., 2018). However, on the other hand, recent studies provide evidence for the role of metabolic perturbations in accelerated aging (Spinelli et al., 2020). These transformations, following either way, involve diverse interactions between molecular players of aging, metabolism, and redox biology (including mitochondria fitness, Ca^2+^ signaling, and bioenergetics); all encrypted in the genomic sequence. Accumulation of irreversible genomic changes over a long time then leads to the onset and progression of cancer. Cancer cells have been shown to operate with reengineered metabolic processes to satisfy their surplus needs during uncontrolled proliferation (Liu et al., 2022). Therefore, aging, metabolic changes, and cancer exist as a network of crossroads (Tidwell et al., 2017; Golubev and Anisimov, 2019; Poljsak et al., 2019). These broadly categorized pathologies share common genomic signatures that further strengthen the link between aging, metabolic disorders, and cancer (Aunan et al., 2017; Lacroix et al., 2020). Along with metabolic alterations, occurrence of aberrant mutations in the mitochondrial genome is also a common characteristic of aging and cancer (Smith et al., 2022). Therefore, it becomes important to uncover the contribution of genomic changes in the context of these cellular health states and the sequential order that defines these states, if any. In line with this, the original research articles and reviews published in the present Research Topic focus on genomic alteration landscapes of aging, metabolic disorders and cancer, the existing and emerging overlaps, and its clinical importance for therapeutic interventions (Figure 1).

The role of autophagy has been recently established in the pathogenesis of aortic dissection; however, the complete molecular mechanism has not been uncovered yet. In an original report, Huang et al. has studied the correlation between the family of ULK (UNC51-like enzymes) genes and the age of first onset of type B aortic dissection (TBAD). The authors analyzed the genome of 159 TBAD patients from Chinese population. A pool of 1,180,097 SNPs was included. Among the different ULK genes, only ULK4 was found to be significantly associated with the first onset age. They concluded that high level of ULK4 gene expression was related to delayed onset of TBAD among these patients. Further experimental validation of these findings can suggest ULK4 to be a diagnostic target for TBAD.

Telomere shortening is one of the important hallmarks of cellular senescence, however, telomere length related cellular senescence has been shown to have varying effects in different cancers. To delineate this paradoxical relationship, Son et al. made use of 42 telomere length associated SNPs, and performed Mendelian randomization analysis to explore the causal relationship between telomere length, skin aging and the susceptibility risk of different skin cancer types. The authors found that telomere shortening can promote aging of the skin and reduce the risk of cutaneous melanoma and non-melanoma skin cancer.

As mentioned before, cellular senescence has often been correlated to oncogenic activation and tumor suppression leading to cancer development. In line with this, Dai et al. has constructed a prognostic risk score signature using the senescence related genes differentially expressed in gastric cancer samples. A total of 135 such genes were identified with significant dys-regulation. Integration of survival data associated 24 of these genes with gastric cancer prognosis. Patients with high expression of SMARCA4 (gene with highest mutational frequency) were associated with higher overall survival and progression-free survival. A total of 11 genes were then identified using LASSO Cox regression analysis to develop the prognostic risk score signature. Testing using an independent data showed that this signature could accurately distinguish low-risk and high-risk samples. The authors further showed that the low-risk score group was also more susceptible to chemotherapy and immunotherapy, and hence can be used for better decision making for treatment to be given. Another study by Dai et al. on similar grounds looked into the cellular senescence related genes that can be used for prediction of prognosis and immunotherapy response in colon cancer patients. Dong et al. and Sun et al. also reported a senescence-related prognostic model that has been shown to predict the prognosis, immunotherapeutic response, and identify potential drug targets for colorectal and hepatocellular carcinoma patients, respectively. These studies show the potential of using huge amount of publicly available clinical data for learning and developing predictive models to design personalized treatment regimen for cancer patients.

Intracellular calcium levels play an important role in homeostasis and various cell signalling processes. Dysregulated levels of calcium have been shown to be remarkably associated with cancer growth, angiogenesis, and metastasis. Elevated serum calcium level is a proposed diagnostic marker for head and neck malignancy. In association with this, Hegde et al. carried out *in silico* analysis to demonstrate the role of store-operated calcium channels in regular mitochondrial function, and further suggest that alteration in these calcium channels might be a predictive and prognostic marker for head and neck squamous cell cancer patients.

Though cancer in general is thought to arise from accumulation of somatic mutations, they do have a substantial hereditary component. To look at the contribution of the pathogenic germline variants in the development of bowel cancer in Chinese population, Xie et al. analyzed the mutation profile of 573 patients accounting for various stages of bowel/colorectal cancer. The profiled germline mutations were categorized as pathogenic, likely-pathogenic and non-pathogenic. Some rare germline alterations in genes like ANCD2, CDH1, and FLCN were also observed. The other germline mutations were enriched in genes involved in DNA-damage repair and homologous recombination. Patients carrying germline mutations also showed a distinctive somatic mutation profile and tumor mutation burden, which also affected the overall survival of these patients. This study provides an assessment of a wider range of susceptibility genes in Chinese bowel/colorectal patients.

Along with accumulating genetic mutations, cancer cells also reprogram the other biochemical processes to generate conditions favorable for sustenance and continuous proliferation. Metabolic reprogramming to switch from oxidative phosphorylation to aerobic glycolysis is one of the major hallmarks of cancer. In this Research Topic of articles, Sharma et al. has presented a comprehensive review highlighting the role of hypoxia-inducible factor-1 (HIF-1) in imparting aggressive behavior in cancer cells through hypoxic glycolysis, and novel therapeutic strategies currently available for targeting HIF-1 in cancer.

Not only gene expression, but its regulation by non-coding RNAs like miRNA also plays a crucial role in onset and progression of various diseases. A review article from Chhichholiya et al. gives information about the reported single nucleotide polymorphisms in miRNA(s) and their target sequences known to be involved in cancer and diabetic pathologies.

In summary, the present Research Topic gathers original research and comprehensive reviews highlighting the genomic factors behind aging, metabolic perturbations and cancer. These studies confirm the interconnecting links between these pathologies, and the need to understand these to identify the cross-points that can be further explored for diagnosis, prognosis and other therapeutic interventions.
